# A Computer-Simulation Study on the Effects of MRI Voxel Dimensions on Carotid Plaque Lipid-Core and Fibrous Cap Segmentation and Stress Modeling

**DOI:** 10.1371/journal.pone.0123031

**Published:** 2015-04-09

**Authors:** Harm A. Nieuwstadt, Zaid A. M. Kassar, Aad van der Lugt, Marcel Breeuwer, Anton F. W. van der Steen, Jolanda J. Wentzel, Frank J. H. Gijsen

**Affiliations:** 1 Department of Biomedical Engineering, Erasmus MC, Rotterdam, the Netherlands; 2 Department of Radiology, Erasmus MC, Rotterdam, the Netherlands; 3 Philips Healthcare, Best, the Netherlands; 4 Department of Biomedical Engineering, Eindhoven University of Technology, Eindhoven, the Netherlands; 5 Department of Imaging Science and Technology, Delft University of Technology, Delft, the Netherlands; Shenzhen Institutes of Advanced Technology, CHINA

## Abstract

**Background:**

The benefits of a decreased slice thickness and/or in-plane voxel size in carotid MRI for atherosclerotic plaque component quantification accuracy and biomechanical peak cap stress analysis have not yet been investigated in detail because of practical limitations.

**Methods:**

In order to provide a methodology that allows such an investigation in detail, numerical simulations of a T1-weighted, contrast-enhanced, 2D MRI sequence were employed. Both the slice thickness (2 mm, 1 mm, and 0.5 mm) and the in plane acquired voxel size (0.62x0.62 mm^2^ and 0.31x0.31 mm^2^) were varied. This virtual MRI approach was applied to 8 histology-based 3D patient carotid atherosclerotic plaque models.

**Results:**

A decreased slice thickness did not result in major improvements in lumen, vessel wall, and lipid-rich necrotic core size measurements. At 0.62x0.62 mm^2^ in-plane, only a 0.5 mm slice thickness resulted in improved minimum fibrous cap thickness measurements (a 2–3 fold reduction in measurement error) and only marginally improved peak cap stress computations. Acquiring voxels of 0.31x0.31 mm^2^ in-plane, however, led to either similar or significantly larger improvements in plaque component quantification and computed peak cap stress.

**Conclusions:**

This study provides evidence that for currently-used 2D carotid MRI protocols, a decreased slice thickness might not be more beneficial for plaque measurement accuracy than a decreased in-plane voxel size. The MRI simulations performed indicate that not a reduced slice thickness (i.e. more isotropic imaging), but the acquisition of anisotropic voxels with a relatively smaller in-plane voxel size could improve carotid plaque quantification and computed peak cap stress accuracy.

## Introduction

Carotid magnetic resonance imaging (MRI) is an established modality to image atherosclerotic plaques at the common carotid artery bifurcation [1 *–* 3]. MRI is the only currently available, noninvasive modality to visualize the plaque fibrous cap (FC) and components such as the lipid-rich necrotic core (LRNC) with high contrast, allowing for plaque segmentation [4 *–* 6]. Segmentation data can be used to quantify plaque components and to compute the peak cap stress—a biomechanical marker for rupture risk—via finite element analysis (FEA) [7–10].

While three-dimensional (3D) carotid MRI protocols with isotropic spatial resolution have recently been introduced [11 *–* 14], the majority of current clinical protocols remain slice-selective, two-dimensional (2D) sequences [15]. In 2D protocols, anisotropic voxels are acquired with a slice thickness larger than the in-plane acquired voxel size. A slice thickness of 2–3 mm is most commonly used [3,16,17]. Acquiring anisotropic voxels can improve the signal-to-noise ratio (SNR) and/or decrease the total scan time while maintaining a small in-plane voxel size to facilitate visualization in a cross-sectional plane [6,18]. It is commonly argued that a decreased slice thickness (or isotropic acquisition) would improve imaging by reducing the influence of axial intravoxel partial volume effects caused by axial morphological variations of a plaque within a slice in the slice-select direction [12,17,19]. However, decreasing the slice thickness requires a sacrifice in SNR and/or scan time, so careful considerations are called for when making such trade-offs [20]. For example, a study by Balu et al. found no difference in measurements of the lumen area, vessel wall area and wall thickness when comparing protocols with slice thicknesses of 0.7 mm (3D, isotropic) and 2 mm (2D, anisotropic) [12]. For more crucial, vulnerable plaque parameters—being LRNC size, minimum FC thickness, and peak cap stress—the potential benefits of a decreased slice thickness in MRI have not been investigated.

The reason why such investigations have not yet been performed lies in the fact that they require a methodology that provides a direct comparison with a ground truth (i.e., the exact underlying geometry) and where the isolated influence of changes in the acquired voxel dimensions can then be studied in detail. A controlled environment would be needed where other parameters (e.g., noise, motion, and image registration) are kept unaltered and where scan duration due to running many protocols do not pose severe practical limitations on patient inclusion. During recent years, numerous studies presented numerical “virtual” MRI is an effective new methodology to achieve such a controlled environment [21–23]. Through modeling MR physics (i.e., solving the Bloch equations) guided by scanner-properties, an input geometry with pre-assigned MR properties (e.g., magnetization, relaxation times), and a pulse sequence, one can computer-simulate an *in vivo* MR image. Due to advances in computer power and the availability of open-source software packages, MRI simulations are increasingly being used to answer clinical image-based questions [23–25].

In a previous study, we performed numerical simulations of a 2D, contrast-enhanced, T1-weighted carotid MRI protocol [25] using the Jülich Extensible MRI Simulator (JEMRIS) [26]. In this current study we adopted a similar approach. We created a set of 3D ground truth carotid plaque models from histological patient data and performed MRI simulations. We focused on slice thickness and intravoxel partial volume effects which, in turn, affect segmentation accuracy. We quantified the impact of a decreased slice thickness on (1) the measurement error of the lumen area, vessel wall area, LRNC area, and minimum FC thickness and (2) the error in computed peak cap stress. To study the combined influence of in-plane resolution and slice thickness, we repeated the aforementioned investigation with a reduced in-plane voxel size.

## Methods

### Ethics statement

This study was approved by the Medical Ethical Committee of the Erasmus Medical Center. Written informed consent was obtained from all subjects.

### Histology

Histological data were used to create a set of sufficiently realistic 3D computer models of carotid plaque geometries to serve as ground truth input sample models for the MRI simulations. Atherosclerotic specimens, obtained at carotid endarterectomy, were decalcified and embedded in paraffin for histological processing. Cross-sectional slices of 5 μm thickness were obtained at 1 mm intervals, and an Elastica van Gieson stain was applied. Histological data from eight (n = 8) patients met our requirements, which were: (1) the presence of at least three successive, largely undeformed and undamaged, cross sections which (2) covered at least one large LRNC with a FC. Micron resolution digitized microscopy images of the histology cross-sections were manually segmented for LRNC and fibrous tissue.

### Geometry Reconstructions

**Fig 1:**
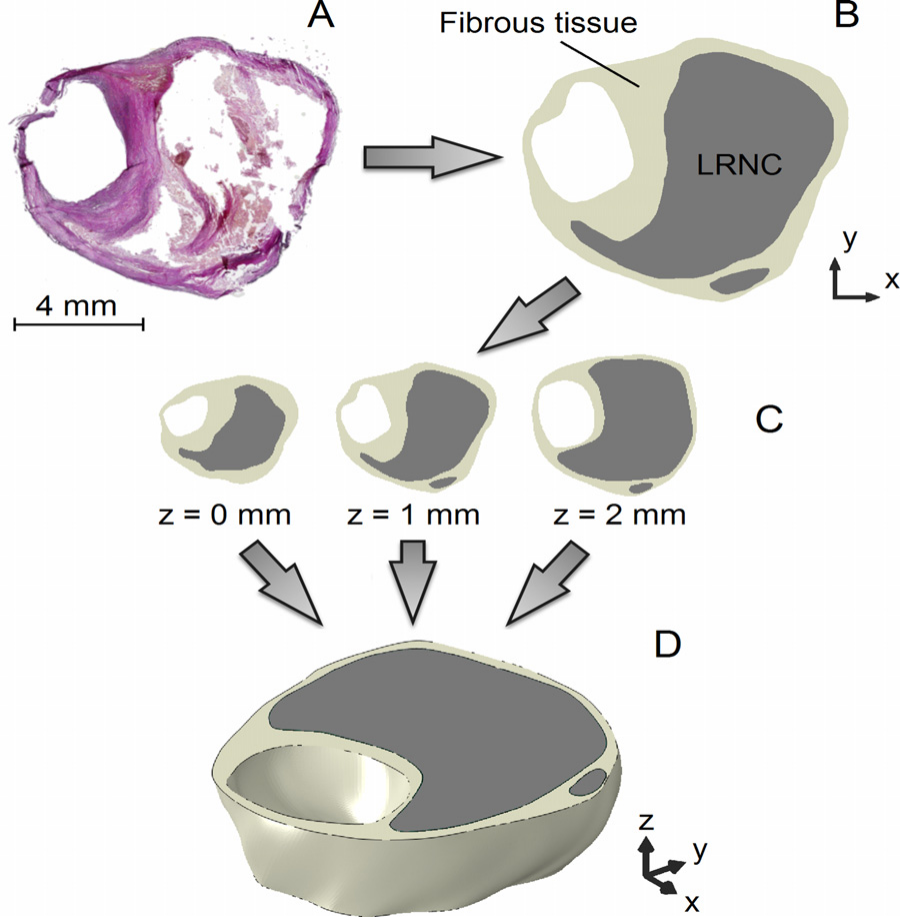
The reconstruction procedure of a 2-mm thick 3D ground truth carotid plaque model illustrated using an example. (A) Histological section, (B) segmentation of the microscopy image, (C) combination of three cross sections, and (D) reconstructed, interpolated, 3D ground truth plaque model.

The eight 3D patient plaque models were constructed by vertically stacking the three histology segmentations with intervals of 1 mm and interpolating the contour data in the axial direction (*z*-direction) with smooth surfaces defined by non-uniform rational basis splines (Gambit, Fluent Inc., ANSYS, Canonsburg, Pennsylvania, USA) [27], as shown in Fig 1. The contours were aligned by the luminal center of gravity. Prior to simulating MRI, the ground truth models were computationally inflated to 100 mmHg using 3D FEA (see section “Finite Element Analysis”). This deformation was applied because the histological sections were not fixated under physiological pressure.

### MRI simulations

A typical clinically applied 3.0T 2D T1-weighted turbo spin-echo, contrast-enhanced, black-blood pulse sequence used to image the FC and LRNC, [16], was implemented in JEMRIS, an open-source numerical Bloch-equation solver [26]. Full details on this specific implementation and an evaluation of *in vivo* MRI simulations have been previously described [25]. The original protocol had an in-plane acquired voxel size of 0.62x0.62 mm^2^ (size adopted in clinical practice) and repetition/echo times of 800 ms/10 ms respectively. Through k-space zero padding, a reconstructed (interpolated) voxel size of 0.31x0.31 mm^2^ was achieved. The simulated pulse sequence was modified through the definition of non-selective radio frequency pulses and the removal of slice-select and spoiler gradients, which resulted in single slice simulations. Three slice thicknesses were simulated: 0.5 mm, 1 mm, and 2 mm, the latter being the slice thickness of the clinically applied protocol. Because the simulated pulse sequence was not slice-selective the input 3D plaque geometries were altered to simulate MRI with a certain slice thickness: for a 2-mm slice, the entire 3D plaque ground truth model, from *z* = 0 mm to *z* = 2 mm, was used as input. For a 1-mm slice, the 3D ground truth model only between *z* = 0.5 mm and *z* = 1.5 mm was used, and for a 0.5-mm slice only between *z* = 0.75 mm and *z* = 1.25 mm. A protocol modification with a doubling of the phase-acquisition steps resulted in a reduced in-plane acquisition voxel size of 0.31x0.31 mm^2^ (0.16x0.16 mm^2^ reconstructed). Hence, a total of 6 scan protocols were simulated in this study: two in-plane acquired voxel sizes (original protocol 0.62x0.62 mm^2^, modified protocol 0.31x0.31 mm^2^) each with three slice thicknesses (2 mm, 1 mm, and 0.5 mm). The smallest simulated acquired voxel size was 0.31x0.31x0.5 mm^3^ (volume of 0.05 mm^3^) which is, currently, far from achievable in a clinical setting. We chose to simulate this voxel size to also add a prospective value to our study. The largest voxel size was 16 times larger, 0.62x0.62x2 mm^3^ (volume of 0.77 mm^3^), and was identical to the voxel size from the original, clinically applied protocol. Noise was superimposed in post-processing to yield an SNR of 16.7 [25]. Because we were interested in *solely* the influence of voxel dimensions we chose specifically to not vary the SNR. Fibrous tissue, LRNC, and the background (sternocleidomastoid muscle) were modeled with T_1_ relaxation times of 680 ms, 1220 ms, and 1412 ms, respectively, and a T_2_ of 50 ms (incorporating gadolinium uptake). Perfect blood signal suppression was presumed and motion effects were not simulated. The simulated MR images were presented in randomized order, on pre-set contrast-brightness settings to an experienced, blinded, MR reader (Z.K.) for manual segmentation. To avert learning-effects, the lower in-plane resolution images (original protocol) were presented first.

### Finite Element Analysis

Tissues were modeled as homogeneous, isotropic, hyperelastic and incompressible using a nonlinear neo-Hookean constitutive model. The material constants were 167 kPa for fibrous tissue and 1 kPa for LRNC [28]. FEA computations were performed in Abaqus (Abaqus Standard, 6.11, Dassault Systèmes Simulia Corp., Providence, Rhode Island, USA). Details on meshing and initial/boundary conditions were described previously [27]. The 3D histology-based ground truth models were deformed to an *in vivo* shape by loading them with a static intraluminal pressure of 100 mmHg before they were used for MRI simulations. The contours from the MR reader segmentations on the single-slice images were converted to 2D models (plane strain formulation). For the stress computations both the 3D (ground truth) and 2D (MRI segmentations) models were loaded with a systolic pressure of 125 mmHg. The initial stresses present in the MRI segmentation models were computed with the backward incremental method [29].

### Analysis

We first studied the geometries of the ground truth models. Three metrics for axial morphological variations in a given slice thickness were defined: (1) the relative area difference between the top and bottom cross sections, (2) the relative nonoverlapping area between those same top and bottom cross sections, and (3) the maximum in-plane (*x*, *y*) shift of the center of gravity. The latter was applied to only the LRNC because the models were defined with an axially aligned luminal center of gravity. For each metric, the absolute value was taken. We then performed the MRI simulations and obtained the lumen area, vessel wall area, LRNC area, minimum FC thickness and peak cap stress from the segmentations. To allow a comparison of a 2D segmentation with the underlying 3D ground truth we normalized for slice thickness by using the ground truth slice averaged area (i.e., volume within the simulated 3D ground truth slice divided by its thickness) instead of volumes. The minimum FC thickness was defined as the shortest distance between LRNC tissue and the lumen. The maximum principal stress was used as the scalar stress measure. For all the aforementioned parameters the relative error was computed with respect to the ground truth value, and a paired Student's t-test was applied to test for statistically significant differences in the mean (significant if *p*<0.05). Data are shown as the mean ± standard deviation.

## Results

### Ground truth plaque models

**Fig 2:**
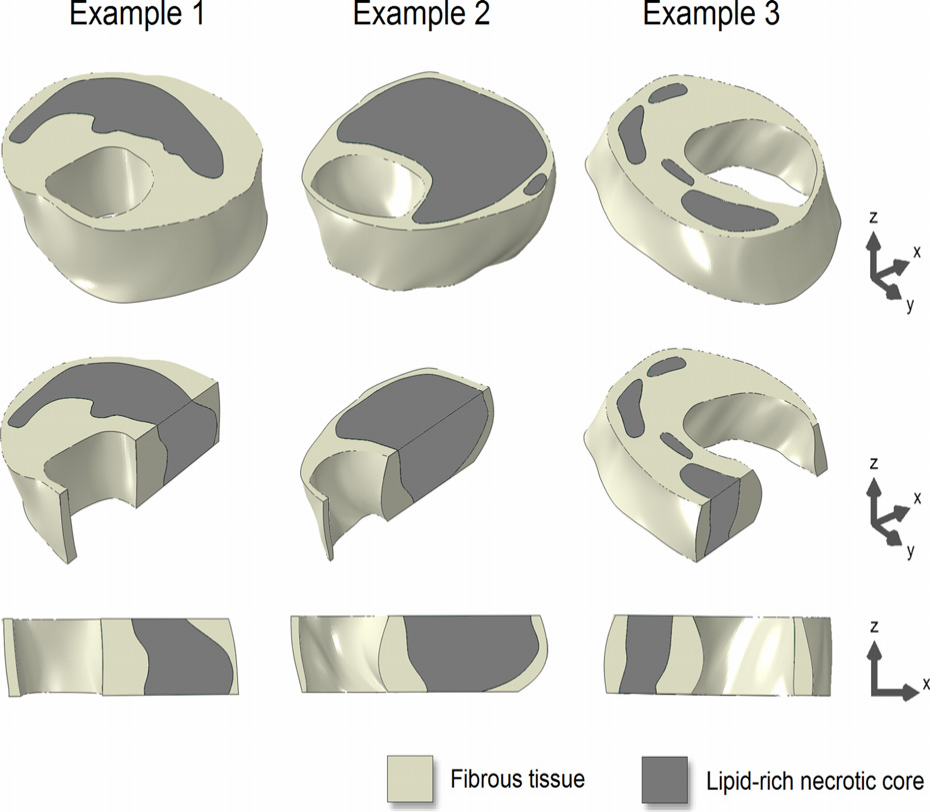
Three examples of the 3D ground truth plaque models (top row). Longitudinal cross-sectional views (middle and bottom rows) illustrate axial morphological variations.

**Fig 3:**
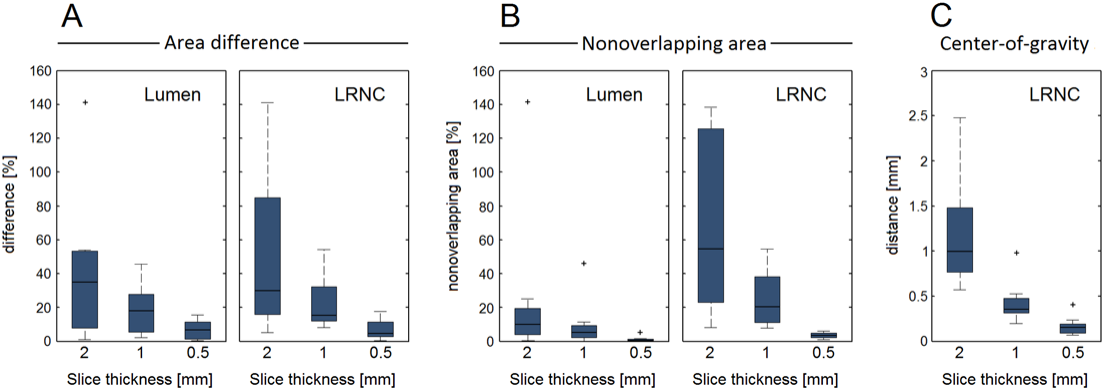
Three metrics quantify the axial geometrical variations in the ground truth models as a function of slice thickness. (A) Relative area difference between slice top and bottom cross sections for lumen and LRNC, (B) relative nonoverlapping area between slice top and bottom cross sections for lumen and LRNC, and (C) maximum in-plane shift of the center of gravity within the slice (C). Whiskers in the box plots represent maximum/minimum data points not considering outliers, which are marked by plus (+) symbols.

Three examples of the 3D ground truth plaque models are shown in Fig 2. All eight models covered a wide range of plaque dimensions. For the 2-mm models, the slice-averaged area for the lumen was 13.4 ± 6.6 mm^2^ (range 5.8–24.9 mm^2^), for the vessel wall 38.6 ± 11.5 mm^2^ (range 25.2–57.4 mm^2^), and for the LRNC 15.8 ± 9.7 mm^2^ (range 6.0–34.6 mm^2^). The minimum FC thickness was 0.27 ± 0.20 mm (range 0.10–0.67 mm). In Fig 3, the three metrics for axial morphological variations (area difference, nonoverlapping area, and center of gravity) are shown in box plots as a function of the slice thickness.

### Plaque component measurements

**Fig 4:**
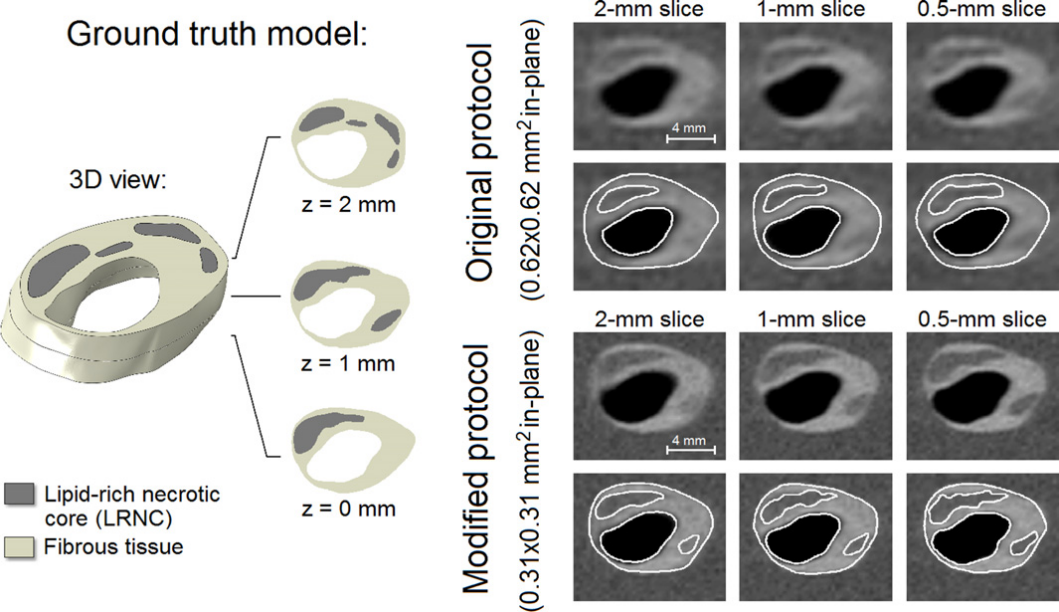
An example of a 3D ground truth input model (left) with its 6 simulated in vivo carotid MR images with different voxel dimensions and segmentation (right). Axial domain of the simulated geometry for a 2-mm slice: z = 0 to 2 mm, for a 1-mm slice: z = 0.5 to 1.5 mm, and for a 0.5-mm slice: z = 0.75 to 1.25 mm.

The example in Fig 4 shows a 3D ground truth plaque model, the simulated *in vivo* carotid MR images, and their segmentations. The ground truth model encompassed one main LRNC covering the entire 2-mm axial distance and various smaller LRNCs at *z* = 1 mm and *z* = 2 mm. Segmentation inaccuracies appeared to be mostly attributable to the limited in-plane resolution, not the slice thickness. The modified MRI protocol (0.31x0.31 mm^2^ in-plane) provided more in-plane detail which resulted in (1) a more accurate segmentation, (2) the positive identification of one additional smaller LRNC at *z* = 1 mm, and (3) more profound axial partial volume effects, making delineation accuracy more dependent on slice thickness. Due to reduced axial partial volume effects when the slice thickness was decreased, the contrast between the FC and LRNC tissues locally increased, improving FC visualization.

**Fig 5:**
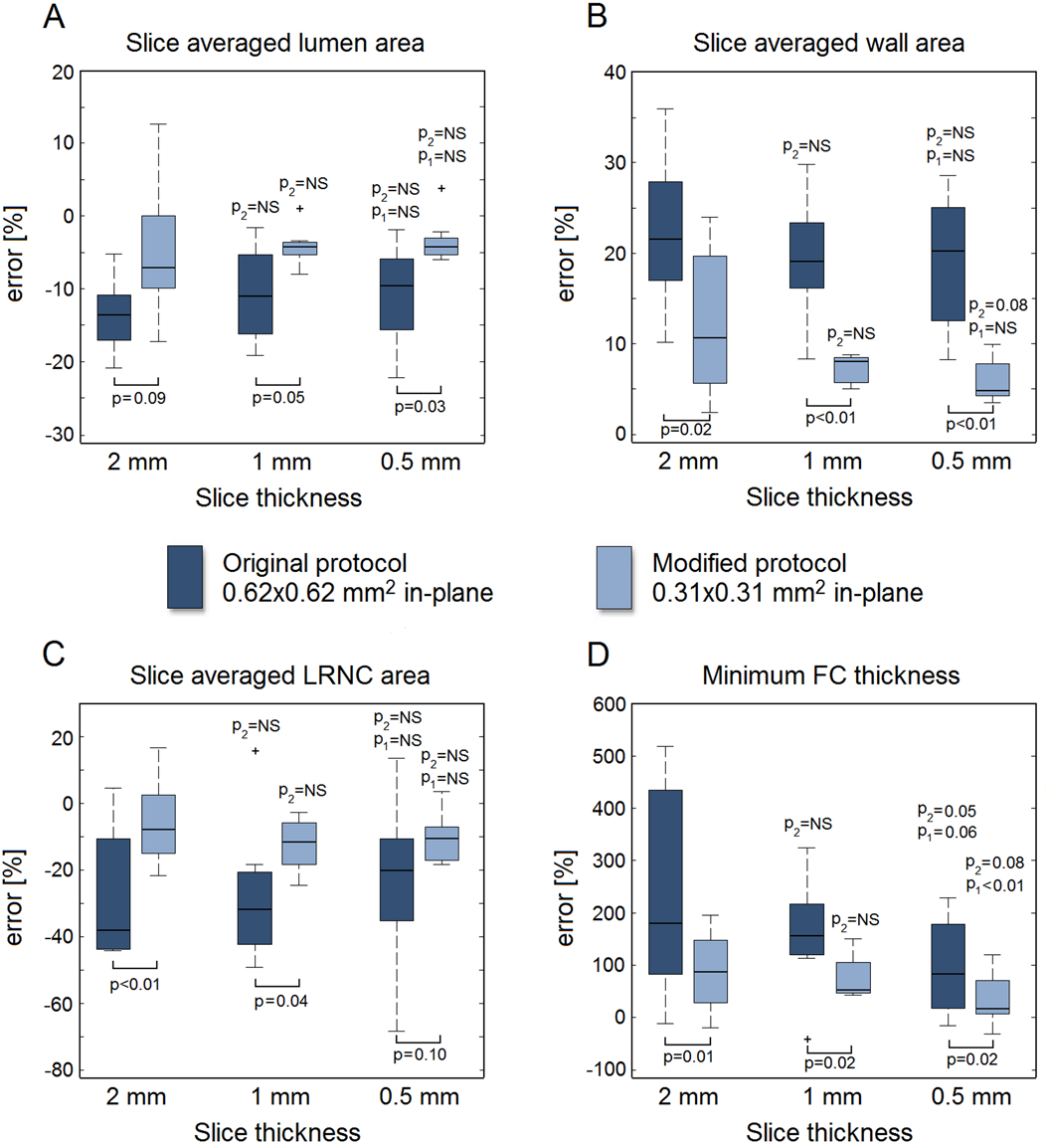
Relative error in measured value with respect to the ground truth for the geometrical parameters studied: (A) lumen area, (B) wall area, (C) LRNC area, and (D) minimum FC thickness. Whiskers in the box plots represent maximum/minimum data points not considering outliers, which are marked by plus (+) symbols. The p-values with respect to the 2-mm and 1-mm slice data are indicated with p2 and p1, respectively. A p-value is denoted as NS (not significant) if p>0.10.

For all eight models, the measurement errors of all geometrical parameters for both in-plane voxel sizes and each slice thickness are shown in box plots in Fig 5. The lumen and LRNC areas were, on average, underestimated while the vessel wall area and minimum FC thickness were overestimated. When measuring the lumen area, vessel wall area, and LRNC area, no major improvements were observed when the slice thickness was decreased (for 0.62x0.62 mm^2^ in-plane). On the other hand, we found larger, statistically significant, improvements when decreasing the in-plane voxel size for any given slice thickness, as well as substantial reductions in error spread (i.e., increased precision) for all geometrical parameters. The minimum FC thickness was the only parameter for which the error significantly improved when only the slice thickness was reduced. This occurred for a 0.5-mm slice thickness versus a 2-mm slice for the original protocol (*p* = 0.05), and versus a 1-mm slice for the modified protocol (*p*<0.01). The measurement error for minimum FC thickness significantly improved from +238% ± 200% for 0.62x0.62x2 mm^3^ voxels to only +35% ± 50% for 0.31x0.31x0.5 mm^3^ voxels (*p*<0.01). We found no significant correlations between the geometrical axial variation metrics in the ground truth models and the measurement errors in MRI.

The fact that a highly anisotropic 0.31x0.31x2 mm^3^ voxel has the same volume (0.19 mm^3^) as a near isotropic 0.62x0.62x0.5 mm^3^ voxel allowed an evaluation regarding voxel anisotropy. LRNC area measurements were far more accurate and precise when acquiring the highly anisotropic voxels instead of near isotropic voxels: an error of -6% ± 13% versus -23% ± 24% (*p* = 0.03). Acquiring the highly anisotropic voxels also improved the mean measurement error of the lumen (-5% anisotropic versus -11% isotropic, *p* = 0.16) and vessel wall (+11% versus +19%, *p* = 0.17) areas. While not statistically significant, the low *p*-values imply trends. For minimum FC thickness, there was no difference (+88% versus +96%, *p* = 0.77), indicating that axial and in-plane FC variations were comparable.

### Plaque stress computations

**Fig 6:**
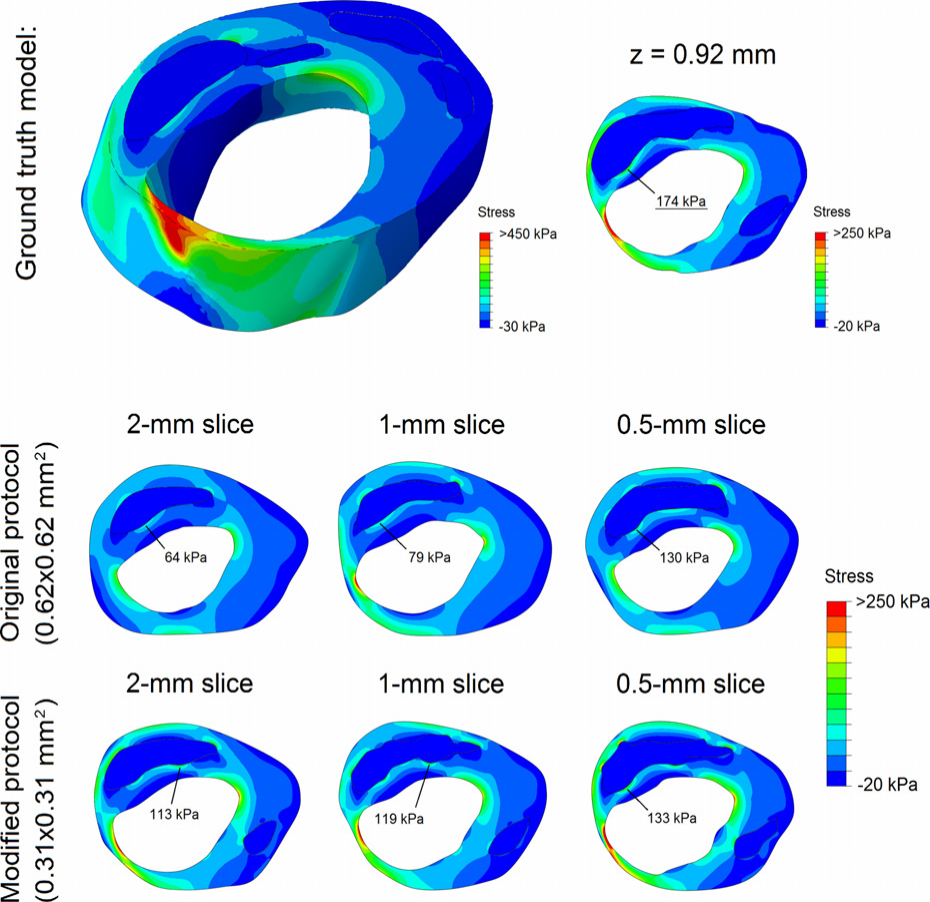
Stress distribution in an example 3D ground truth model (top row) and the stress distributions in its 6 2D MRI segmentation models (middle and bottom rows). The location and magnitude of the peak cap stress is indicated in each model.

The example in Fig 6 shows the stress distributions in a 3D ground truth plaque model and in the corresponding 2D models based on MRI segmentations. In the 3D model a heterogeneous stress distribution was present, with high plaque stresses in the mid-cap (thin) and plaque shoulders (high luminal curvature). The ground truth peak cap stress was 174 kPa (at *z* = 0.92 mm). The MRI-based models exhibited a similar stress distribution, but with a severe peak cap stress underestimation. A thinner segmented FC yielded a higher stress and, in effect, a reduced underestimation. The underestimation of peak cap stress (related to FC thickness overestimation [28]) decreased for a reduced slice thickness and/or a reduced in-plane voxel size.

**Fig 7:**
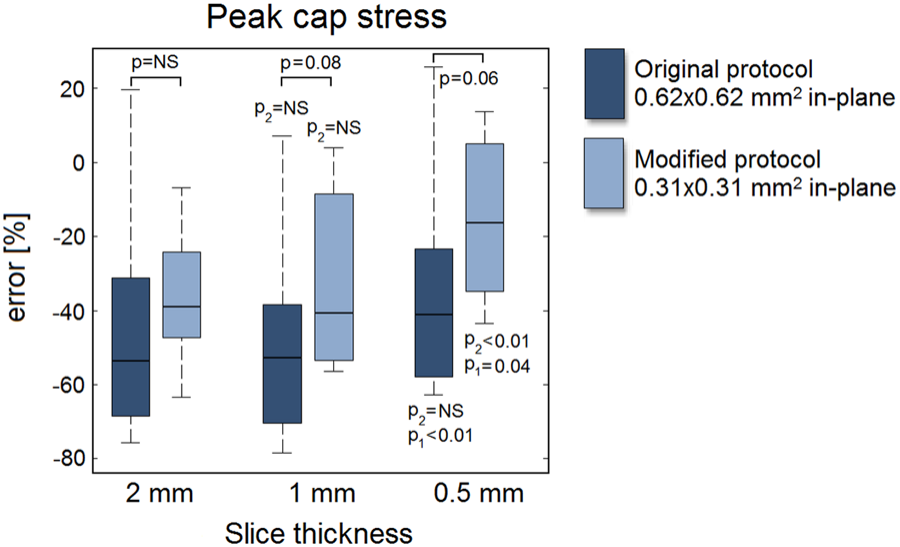
Relative error in the MRI segmentation model predicted peak cap stress with respect to the ground truth peak cap stress as a function of slice thickness. For details, see caption of Fig 5.

For all eight models, the error of the MRI segmentation model peak cap stress is shown in Fig 7 for both the original (0.62x0.62 mm^2^) and the modified protocol (0.31x0.31 mm^2^) as a function of the slice thickness. The peak cap stress was severely underestimated, with a large imprecision (i.e., large error spread). The interquartile ranges indicate no improvements in precision for a decreased slice thickness or in-plane voxel size. A decreased slice thickness only improved the mean error when 0.5-mm slices were acquired, while a reduced in-plane voxel size (for any given slice thickness) always resulted in larger improvements. The low *p*-values (*p* = 0.08 for a 1-mm slice and *p* = 0.06 for a 0.5-mm slice) indicate trends. The smallest voxel size (0.31x0.31x0.5 mm^3^) yielded an error of -15% ± 22% versus an error of -45% ± 32% for the largest voxel size (0.62x0.62x2 mm^3^) (*p*<0.01). Interestingly, acquiring highly anisotropic voxels (0.31x0.31x2 mm^3^) instead of near isotropic voxels (0.62x0.62x0.5 mm^3^) with the same volume had little effect on the peak cap stress error (-36% ± 18% anisotropic versus -35% ± 30% isotropic, *p* = 0.89).

## Discussion

In this study, we investigated the degree to which axial intravoxel partial volume effects, associated with acquiring a specific slice thickness, contribute to errors in atherosclerotic plaque component measurements and peak cap stress computations in carotid MRI. A simulated, virtual MRI approach allowed direct quantification of measurement error in a controlled environment where only the voxel dimensions were varied.

### General observations

Using eight 3D plaque models created from three stacked consecutive histological cross-sections and performing single-slice MRI simulations, we found that for an in-plane acquired voxel size of 0.62x0.62 mm^2^, a decreased slice thickness did not significantly improve measurements of the lumen, vessel wall and LRNC size, but it did have a beneficial effect on the accuracy of minimum FC thickness measurements. Note that we did not simulate any localized scan plane angulations. Furthermore, only a 0.5-mm slice led to a relatively marginal improvement in the error in computed peak cap stress. A reduction in the in-plane voxel size to 0.31x0.31 mm^2^, however, led to similar or often larger improvements. LRNC measurements improved when anisotropic voxels were acquired instead of isotropic voxels of the same volume (error of -6% ± 13% versus -23% ± 24%, *p* = 0.03). Similar trends were observed for the other parameters studied. Our findings provide evidence that current 2D carotid MRI protocols for plaque quantification appropriately sacrifice axial resolution to reduce scan time and/or noise. The commonly used argument that a standard 2-mm slice thickness limits imaging therefore only applies to small, localized features such as the FC. Consequently, 3D carotid MRI protocols could be modified by reducing the slice-select phase-encoding steps (i.e., transitioning from isotropic to anisotropic voxels), thus reducing scan time. We confirmed the reports of Balu et al. with regard to the unimproved vessel wall and lumen measurements [11,12], and, by virtue of our simulation methodology, studied more crucial, vulnerable plaque parameters. A ground truth comparison as employed in our study allowed a quantification of measurement accuracy, the absence of which was a limitation in most previous studies which only assessed reproducibility [9,11,12,18,30]. The observed overestimations (wall area and FC thickness) and underestimations (lumen area, LRNC area, and peak cap stress) were in line with previous reports [25,28,31–33]. A recent study by van Wijk et al., also found that higher voxel anisotropy improved wall measurements [18]. In a previous study, we reported the large inaccuracy in minimum FC measurements [25], while assuming a uniform axial morphology within a slice. The findings from the present study show that intraslice axial FC variations lead to a much larger inaccuracy in measured FC thickness than previously reported [25].

Axial variations rapidly decreased when the slice thickness was reduced. Although indicative, the axial variation metrics were quite strict when linked to MRI segmentation because they only use data on the axial boundaries of a slice. Gradual intensity changes due to partial volume effects in an MRI slice will lead to a correct ‘slice-averaged’ segmentation, and thus to a relatively precise measurement of the component volume. Indeed, with regard to MRI slice thickness, volumetric plaque components are inherently more forgiving than, for instance, minimum FC thickness, which is a very localized parameter both in-plane and axially. The lack of any correlation between the axial variation metrics and measurement errors also suggests that segmentation accuracy is more influenced by the in-plane voxel size than by the slice thickness.

### Clinical implications

In clinical practice, alterations in voxel dimensions affect scan time and SNR. A decrease in voxel size would either result in an increased scan time, a lower SNR, or a combination of both. Scan time and SNR were not the focus of this study and therefore not investigated. A trade-off can easily become more complicated than the mere application of the standard SNR equation when considering, for example, 3D versus 2D protocols [34], motion artifacts [35], and imperfect black-blood signal suppression [30,36]. Note that we found that, even with an *unaltered* SNR, a decreased slice thickness was often not beneficial. In a previous study, we explored the trade-off between scan time, SNR and in-plane resolution, and found that SNR was less limiting than the resolution for manual segmentation [25]. However, Rhonen et al. investigated the effects of SNR and in-plane resolution in an *ex vivo* study using thin histological slices, and concluded that SNR had a large impact on automated tissue classification [20]. While our study provides relevant data, the true clinical benefit when trading-off voxel dimensions against scan time, noise, motion-artifacts and blood signal suppression needs to be investigated in further studies [31]. Additionally, in our study we did not focus on the bifurcation, and we did not incorporate angulations of the simulated vessels. This makes our findings only applicable to fairly straight vessels or an MRI acquisition with an imposed oblique scan plane orientation in the direction of the vessel at the plaque location. In case of large vessel angulations or bifurcations, the use of 3D isotropic imaging could be more beneficial. In especially elderly patients, the left and right carotid arteries tend to not be parallel. Isotropic imaging could also be beneficial when imaging certain other plaque components such as small calcifications. We could not investigate this in this study due to decalcification of the histological specimens. We purposely imposed a relatively high SNR to create MR images that on the one hand were as realistic as possible, but in which, on the other hand, noise would not be the weakest link [25]. We restricted ourselves by focusing on only partial volume effects caused by finite voxel dimensions, because these effects are a critical and often addressed (but insufficiently studied) issue in carotid MRI studies [10,12,17,28,34,37]. We did therefore not study additional possible benefits of a decreased slice thickness in clinical practice such as improved axial image matching in longitudinal studies, improved retrospective multi-planar reformatting [11,12], easier registration to histology slices [17,38], and a decreased sensitivity to a localized oblique scan plane orientation [32]. A reduction in slice thickness would serve a double benefit for plaque FEA since it would also increase the axial sampling resolution for 3D multislice-based plaque FEA [27].

### Study limitations

Eight representative carotid plaques were used. From a statistical point of view our study was exploratory: the sample size was not sufficiently large to corroborate the statistical significance of relatively small differences between means. Nevertheless, such small changes are immaterial for practical applications, especially when considered in conjunction with the observed large spread in errors. Intraplaque hemorrhage was absent in the histological sections, and decalcification inhibited the inclusion of calcifications in the ground truth models. For small calcifications, isotropic imaging can be beneficial [11,12]. The 3D ground truth models were created by interpolating histology slices which had a 1-mm axial spacing. This is a limitation considering the fact that the MRI slice thickness was in the same order of magnitude. However, the examples shown in Figs 1, 2 and 4 indicate the presence of axial variations on sub-millimeter scales (due to the 3D spline interpolation). In addition, the examples do not evidence critical axial under sampling. By aligning the luminal center of gravity of the histological sections, we assumed no oblique scan plane orientation at the slice of interest (for details, see [32]). For the MRI, the imposed SNR was relatively high, motion was neglected, and homogeneous components were used. These factors, when combined, make the errors we report in this study representative for a best-case imaging scenario. Only single-slice simulations were performed with uniform axial excitation, neglecting influences from adjacent slices. This choice was made because such influences (e.g., cross talk in 2D sequences or point spread function effects in the slice-select phase direction in 3D sequences) can be highly protocol-specific. For a detailed discussion regarding the MRI simulations using JEMRIS, we refer to our previous work [25]. Residual stresses, heterogeneity and collagen/elastin fiber directionality were not included in the biomechanical models [39], but this did not compromise the comparisons which involved solely geometrical differences.

## Conclusions

This simulation study provides evidence that in the absence of intraslice vessel obliquity, measurements of the lumen, vessel wall or LRNC size in carotid MRI do not majorly improve when decreasing the slice thickness, even if the SNR remains unaltered. For minimum FC thickness and the closely related peak cap stress magnitude, a decreased slice thickness was beneficial, but not more than a decreased in-plane voxel size. Our simulations provide evidence that in certain cases, not a decreased slice thickness, but the acquisition of anisotropic voxels that can improve lumen, LRNC, and FC thickness quantification and computed peak cap stress accuracy in a clinical setting. The presumed benefit of 3D isotropic voxel acquisition in other cases, such as in the presence of large vessel angulations, bifurcations, or when imaging small plaque components such as calcifications, needs to be quantified in future studies.

## Supporting Information

S1 Fig The other five 3D plaque models used in this study. 3D models (top row) and their longitudinal cross-sectional views (middle and bottom rows) illustrate axial morphological variations.
(TIF)
