# Supplementary figures and images for: A Computer-Simulation Study on the Effects of MRI Voxel Dimensions on Carotid Plaque Lipid-Core and Fibrous Cap Segmentation and Stress Modeling
Source: PLoS One. 2015 Apr 9;10(4):e0123031. doi: 10.1371/journal.pone.0123031 (PMC4391711; doi:10.1371/journal.pone.0123031)

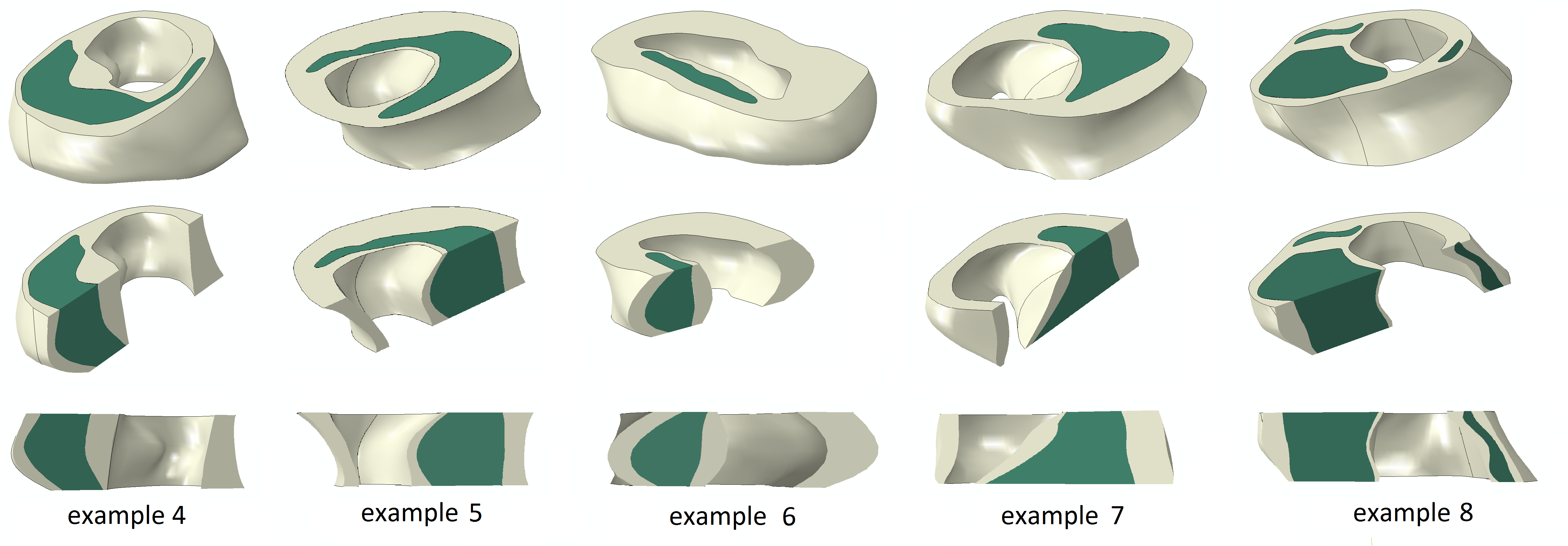

Supplement: S1 Fig — 3D models (top row) and their longitudinal cross-sectional views (middle and bottom rows) illustrate axial morphological variations. (TIF) [file pone.0123031.s001.tif]
